# Endoscopic ultrasound-guided gallbladder drainage using a forward-viewing echoendoscope after Billroth II gastrectomy

**DOI:** 10.1055/a-2271-4124

**Published:** 2024-03-08

**Authors:** Yuichiro Tozuka, Kazuya Sugimori, Haruo Miwa, Takashi Kaneko, Makoto Ueno, Junji Furuse, Shin Maeda

**Affiliations:** 1 91321 Department of Gastroenterology, Kanagawa Cancer Center, Yokohama, Japan; 2 26437 Gastroenterological Center, Yokohama City University Medical Center, Yokohama, Japan; 3 Department of Gastroenterology, Yokohama City University Graduate School of Medicine, Yokohama, Japan

Endoscopic ultrasound-guided gallbladder drainage (EUS-GBD) is effective for treating cholecystitis 1 2 ; however, its application is challenging in patients with surgically altered anatomy 3 . In such patients, the forward-viewing echoendoscope (FV-EUS) is useful 4 . Here, we report a case of a patient who underwent EUS-GBD using FV-EUS after Billroth II gastrectomy.

An 88-year-old woman with acute cholecystitis secondary to placement of a covered metal stent was admitted to our hospital ( Fig. 1 ). The patient had previously undergone Billroth II gastrectomy for a duodenal ulcer. Initially, she underwent percutaneous transhepatic gallbladder drainage (PTGBD) ( Fig. 2 ), followed by EUS-GBD using FV-EUS, which was conducted for the conversion to internal drainage ( Video 1 ).

**Fig. 1:**
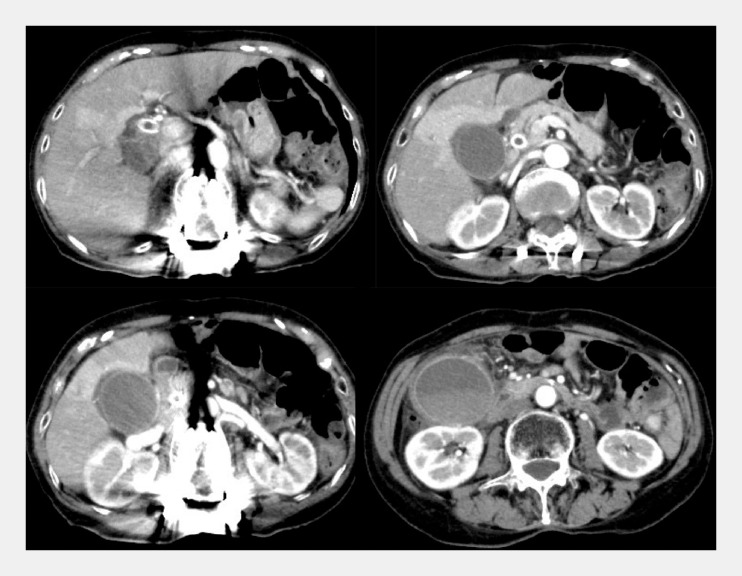
Contrast-enhanced computed tomography performed at the onset of cholecystitis revealed gallbladder swelling. A covered metal stent was placed in the common bile duct.

**Fig. 2:**
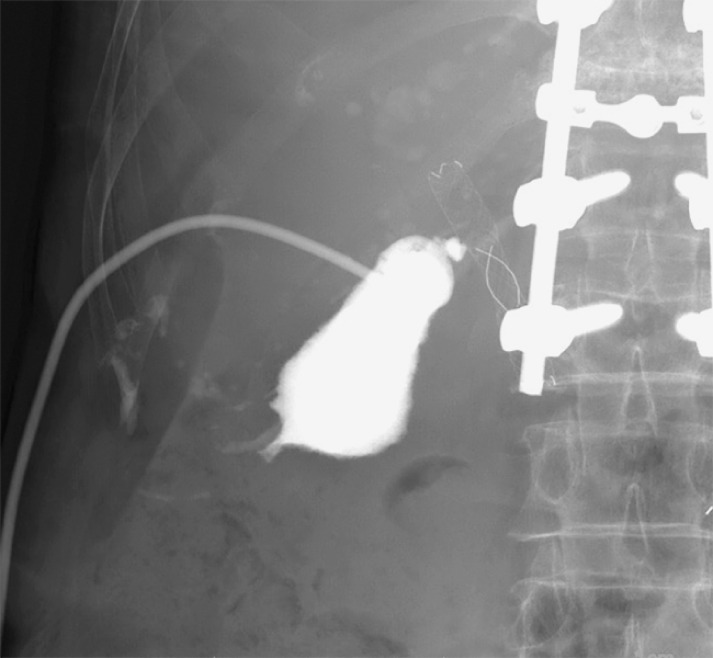
Contrast medium was injected from the percutaneous transhepatic gallbladder drainage; however, the cystic duct was obstructed by the covered metal stent.

Endoscopic ultrasound-guided gallbladder drainage with forward-viewing echoendoscopy was effective in a patient who had undergone Billroth II gastrectomy.Video 1

Before EUS-GBD, a gastroscope (GIF-Q260; Olympus, Tokyo, Japan) was inserted into the afferent loop, and a 0.035-inch guidewire (Boston Scientific Corporation, Marlborough, Massachusetts, USA) was placed. Subsequently, the FV-EUS (TGF-UC260J; Olympus) was advanced to the blind end along the guidewire. In the second part of the duodenum, the gallbladder was observed using ultrasonography after saline injection through the PTGBD. The gallbladder body was punctured with a 19-gauge needle (EZ shot 3; Olympus), and a 0.025-inch guidewire (VisiGlide2; Olympus) was inserted to a sufficient length. The punctured tract was dilated using a 7 Fr mechanical dilator (ES Dilator; Zeon Medical, Tokyo, Japan). A second guidewire was placed using an uneven double-lumen catheter (PIOLAX Inc., Kanagawa, Japan). Finally, a double-pigtail plastic stent (7 Fr and 7 cm; Zimmon Biliary Stent, Wilson Cook Medical, Winston-Salem, North Carolina, USA) was successfully placed in the gallbladder ( Fig. 3 ). With the contrast medium flowing smoothly through the pigtail stent, the PTGBD was removed 6 days after EUS-GBD.

**Fig. 3:**
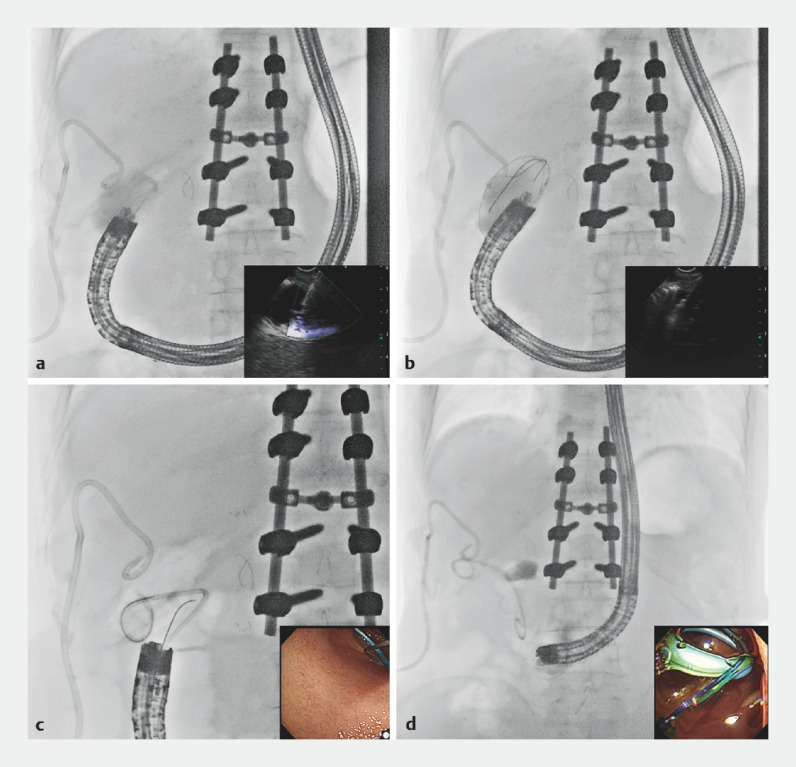
Endoscopic ultrasound-guided gallbladder drainage. **a** The gallbladder body was punctured with a 19-gauge needle. **b** A 0.025-inch guidewire was inserted to a sufficient length. **c,d** A double-pigtail plastic stent was placed.

To the best of our knowledge, this is the first report of EUS-GBD using FV-EUS in a patient with surgically altered anatomy. This procedure holds promise in maintaining quality of life in patients with malignant diseases.

Endoscopy_UCTN_Code_TTT_1AS_2AH

## Notes

Endoscopy E-Videos https://eref.thieme.de/e-videos
*E-Videos* is an open access online section of the journal *Endoscopy* , reporting on interesting cases and new techniques in gastroenterological endoscopy. All papers include a high-quality video and are published with a Creative Commons CC-BY license. Endoscopy E-Videos qualify for HINARI discounts and waivers and eligibility is automatically checked during the submission process. We grant 100% waivers to articles whose corresponding authors are based in Group A countries and 50% waivers to those who are based in Group B countries as classified by Research4Life (see: https://www.research4life.org/access/eligibility/ ). This section has its own submission website at https://mc.manuscriptcentral.com/e-videos .
